# Tissue Reactivity to, and Stability of, Glaucoma Drainage Device Materials Placed Under Rabbit Conjunctiva

**DOI:** 10.1167/tvst.11.4.9

**Published:** 2022-04-11

**Authors:** Kenichi Nakamura, Tomokazu Fujimoto, Miho Okada, Kentaro Maki, Atsushi Shimazaki, Masatomo Kato, Toshihiro Inoue

**Affiliations:** 1 Department of Ophthalmology, Faculty of Life Sciences, Kumamoto University, Kumamoto, Japan; 2 Pharmaceutics and Pharmacology Department, Santen Pharmaceutical Co., Ltd., Nara, Japan

**Keywords:** glaucoma drainage device, conjunctiva, biocompatibility

## Abstract

**Purpose:**

To evaluate tissue reactivity to, and the stability of, glaucoma drainage device materials placed under rabbit conjunctiva in vivo.

**Methods:**

Disks (diameter, 3 mm; thickness, ∼0.3 mm) fabricated from poly(styrene-*block*-isobutylene-*block*-styrene) (SIBS), silicone, stainless-steel, or glutaraldehyde cross-linked collagen (GACLC) were inserted under rabbit conjunctiva. Conjunctival and scleral sections obtained at 4, 8, and 12 weeks after surgery were immunostained for α-smooth muscle actin (SMA). The ratio of the maximum thickness of the α-SMA–positive conjunctiva to the scleral thickness (α-SMA/S ratio) was calculated. The in vivo stability of the drainage devices at 12 weeks after insertion was evaluated.

**Results:**

The mean α-SMA/S ratios of the SIBS and silicone groups were lower than that of the stainless-steel group at 4 weeks after surgery (*P* < 0.05), and that of the SIBS group was lower than that of the GACLC group (*P* < 0.05). The ratios at 8 weeks after surgery were lower in the SIBS and silicone groups than in the GACLC group (*P* < 0.01). The ratios at 12 weeks after surgery were lower in the SIBS and silicone groups than in the GACLC group (*P* < 0.05). The surface areas of GACLC disks explanted from conjunctivae were significantly lower than that of intact disks (*P* < 0.01).

**Conclusions:**

SIBS and silicon were highly biostable and exhibited less tissue reactivity than GACLC in vivo.

**Translational Relevance:**

Comparisons of materials using animal models can predict the clinical stability and safety of such materials in humans.

## Introduction

Glaucoma is an optic neuropathy caused by various factors. Elevated intraocular pressure (IOP) is a major risk factor,^1^^,^^2^ and IOP is the only risk factor proven to be treatable. Medication, laser treatment, and open filtration surgery are used to reduce IOP, and trabeculectomy (a filtration surgery) is widely performed as a glaucoma treatment.^3^ Surgery involving the placement of various drainage devices from the anterior chamber to the subconjunctiva is also employed, especially after failure of trabeculectomy.^4^^–^^7^

Failure of glaucoma filtration surgery is attributable mainly to fibrosis, principally caused by fibroblasts developing in or around the filtering bleb during wound healing.^8^ Although antifibrotic agents such as mitomycin C prevent bleb scarring well and are thus useful during glaucoma filtration surgery,^9^ most surgical failures are attributable to fibrosis despite the use of such agents. As a glaucoma drainage device is a foreign body, the material used for device fabrication must not trigger fibrosis. One such material, poly(styrene-*block*-isobutylene-*block*-styrene) (SIBS), has been used successfully since 2000 to coat cardiovascular stents.^10^ SIBS is a flexible and biostable elastomeric polymer that undergoes minimal biodegradation in vivo.^11^^–^^13^ Recently, the PreserFlo MicroShunt drainage device, fabricated entirely of SIBS, has become available, and IOP-lowering effects have been reported in both clinical and basic research studies.^14^^–^^16^ The PreserFlo MicroShunt is highly biocompatible and would be expected to minimize postoperative fibrosis. Although the inflammatory responses to various materials used to prepare glaucoma drainage devices have been investigated,^11^^,^^17^^–^^19^ the effects on surrounding tissues of most materials used to fabricate modern glaucoma drainage devices have not been examined; such devices include those made from SIBS (the PreserFlo MicroShunt), silicone (large-plate glaucoma implants), stainless steel (the EX-PRESS Glaucoma Filtration Device), and glutaraldehyde cross-linked collagen (GACLC; the XEN Gel Stent). It is important to establish that these materials do not trigger fibrosis, which might cause surgical failure. Here, we report the tissue reactivity and biostability of various glaucoma drainage device materials (including SIBS) placed under rabbit conjunctiva in vivo.

## Materials and Methods

### Materials

Columnar disks (diameter, 3 mm; thickness, ∼0.3 mm) fabricated from SIBS (PreserFlo MicroShunt), silicone, stainless-steel, or GACLC were supplied by Santen Pharmaceutical Co., Ltd. (Osaka, Japan). GACLC was prepared as described in U.S. Patent Number 9113994.

### Implantation of Disks Under the Conjunctiva of Rabbit Eyes

All animal experiments were conducted in accordance with the ARVO Statement for the Use of Animals in Ophthalmic and Vision Research and were approved by the Animal Care and Use Committee of Kumamoto University or Santen Pharmaceutical Co. Ltd. Rabbits were anesthetized with ketamine hydrochloride (50 mg/kg; Daiichi Sankyo Propharma, Tokyo, Japan) and xylazine hydrochloride (10 mg/kg; Bayer HealthCare, Leverkusen, Germany), and the conjunctiva was incised 3 mm distal from the corneoscleral limbi. The conjunctiva was bluntly separated from the sclera using scissors. The disks were inserted into the space thus created. All wounds were sutured in two places with 10-0 nylon.

### Evaluation of the Effects of the Materials on the Conjunctiva

**Figure 1.**
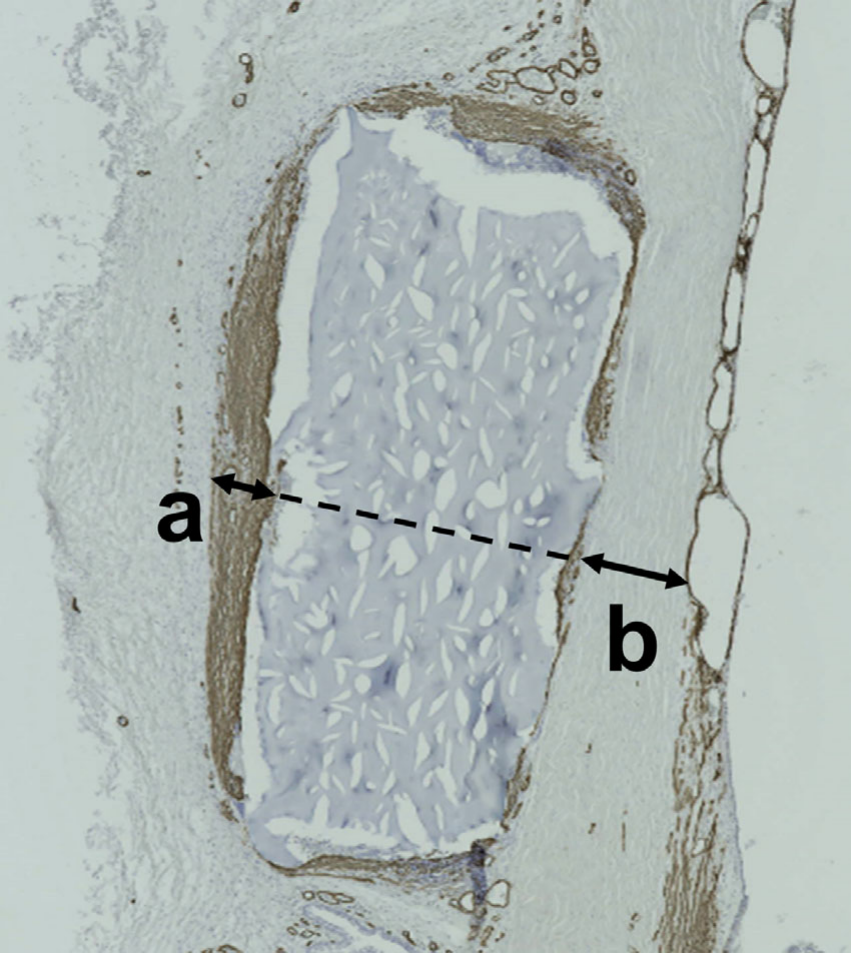
Evaluation of tissue sections. This example was from an eye in the GACLC group. (a) Maximum thickness of the α-SMA–positive part of the conjunctiva on the disk outside the eye. (b) Thickness of the sclera vertical to the same part. The α-SMA/S ratio was obtained by dividing (a) by (b).

SIBS, silicone, stainless-steel, and GACLC disks were inserted under the conjunctiva of rabbit eyes; one disk was inserted into each eyeball. Twelve disks of each material were inserted (48 eyes). We obtained digital photographs of the insertion sites using a COOLPIX S9900 camera (Nikon, Tokyo, Japan) at 4, 8, and 12 weeks after surgery to allow visual assessment, and then we removed the eyes for histological assessment after euthanasia with thiopental sodium (300 mg intravenous; Nipro ES Pharma, Osaka, Japan). Immunostaining was performed as described previously.^16^ Enucleated eyes were fixed in Super Fix (Kurabo, Osaka, Japan) overnight at 4°C and then soaked in 30% (w/v) sucrose (FUJIFILM Wako Pure Chemical Corporation, Osaka, Japan) for 48 hours at 4°C. The eyes were dissected to obtain the entire incisional areas and embedded in an optimal-cutting-temperature compound (Sakura Finetek, Torrance, CA). All tissue samples were sliced vertical to the disk, except those of the stainless-steel group (in which the disks were removed before slicing). Two tissue sections/eye were stained with α-smooth muscle actin (SMA) antibody (1:1,000; Dako, Kyoto, Japan) and counterstained with hematoxylin; another section was stained with collagen type I antibody (1:200; Abcam, Cambridge, UK). The samples were observed under an all-in-one epifluorescence microscope (BZ-X710; Keyence, Osaka, Japan), and photographs of whole sections were created by combining multiple images using BZ-H3A software (Keyence). The ratios of the maximum thickness of the α-SMA–positive conjunctival region to the scleral thickness in the same region (α-SMA/S ratio) were calculated (Fig. 1). At least three eyeballs were evaluated by immunostaining at 4, 8, and 12 weeks after insertion of each material. Two sections of each eye were evaluated; all data were analyzed.

### In Vivo and In Vitro Disk Stability

SIBS, silicone, and GACLC disks were inserted under the conjunctiva of rabbit eyes. Each of the three materials was tested in three eyes. The eyes were removed at 12 weeks after surgery, after the animals had been euthanized with thiopental sodium (300 mg intravenous, Nipro ES Pharma), and the surface areas of explanted and intact disks were measured using a digital microscope (VHX-5000; Keyence) running VHX-H4M software (Keyence). The surface areas of explanted and intact disks were statistically analyzed. Intact disks were soaked in H_2_O_2_ (20% v/v) at 37°C in vitro to mimic in vivo oxidation. Changes in appearance were noted and compared to those of controls soaked in H_2_O at 37°C for 4 weeks using an all-in-one fluorescence microscope (BZ-9000; Keyence); disk photographs were created by combining multiple images using BZ-H2A software.

### Statistical Analysis

Statistical analysis was performed using JMP 8 software (SAS Institute, Cary, NC). The Steel–Dwass test was employed to compare the α-SMA/S ratios among the groups because the ratios were nonparametric and heteroscedastic. The *F*-test was performed followed by Student's *t*-test using SAS 9.4 (SAS Institute) to compare the surface areas of explanted disks. *P* < 0.05 was considered statistically significant. All data are shown as means ± standard deviations.

## Results

### Macroscopic Findings

**Figure 2.**
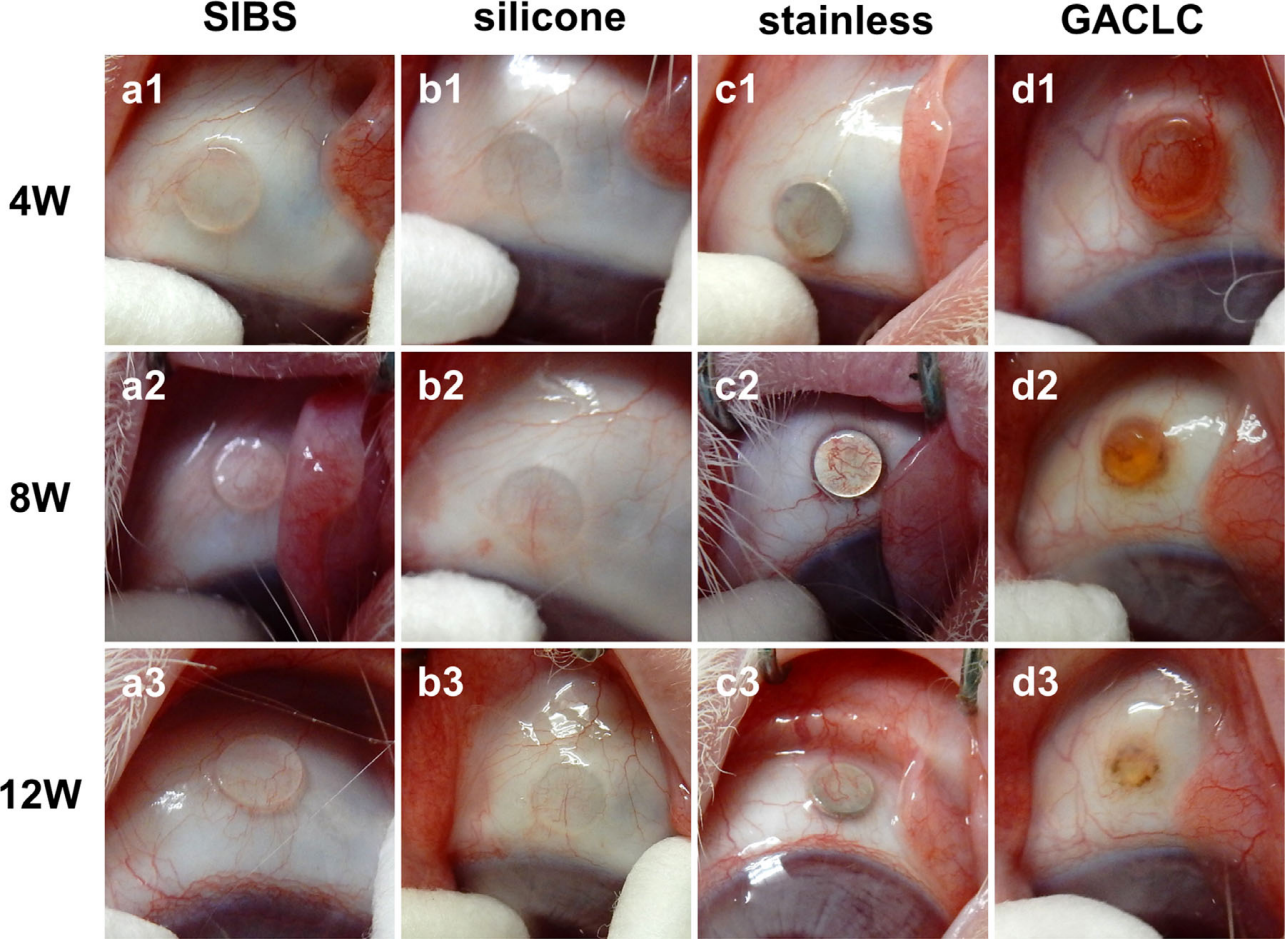
Photographs of conjunctivae after the operation. The columns show the same eyes in the SIBS (a1–a3), silicone (b1–b3), stainless-steel (c1–c3), and GACLC (d1–d3) groups. The rows show the eyes at 4, 8, and 12 weeks after the operation.

Representative conjunctival photographs of the disk insertion sites are shown in Figure 2. At 8 and 12 weeks after the operation, mild hyperemia was observed on the disks in the SIBS and silicone groups, whereas moderate hyperemia was observed on the disk in the stainless-steel group. In the GACLC group, moderate hyperemia was observed on the disk at 4 weeks after the operation, and from 8 to 12 weeks the disk shrank.

### Histologic Findings

**Figure 3.**
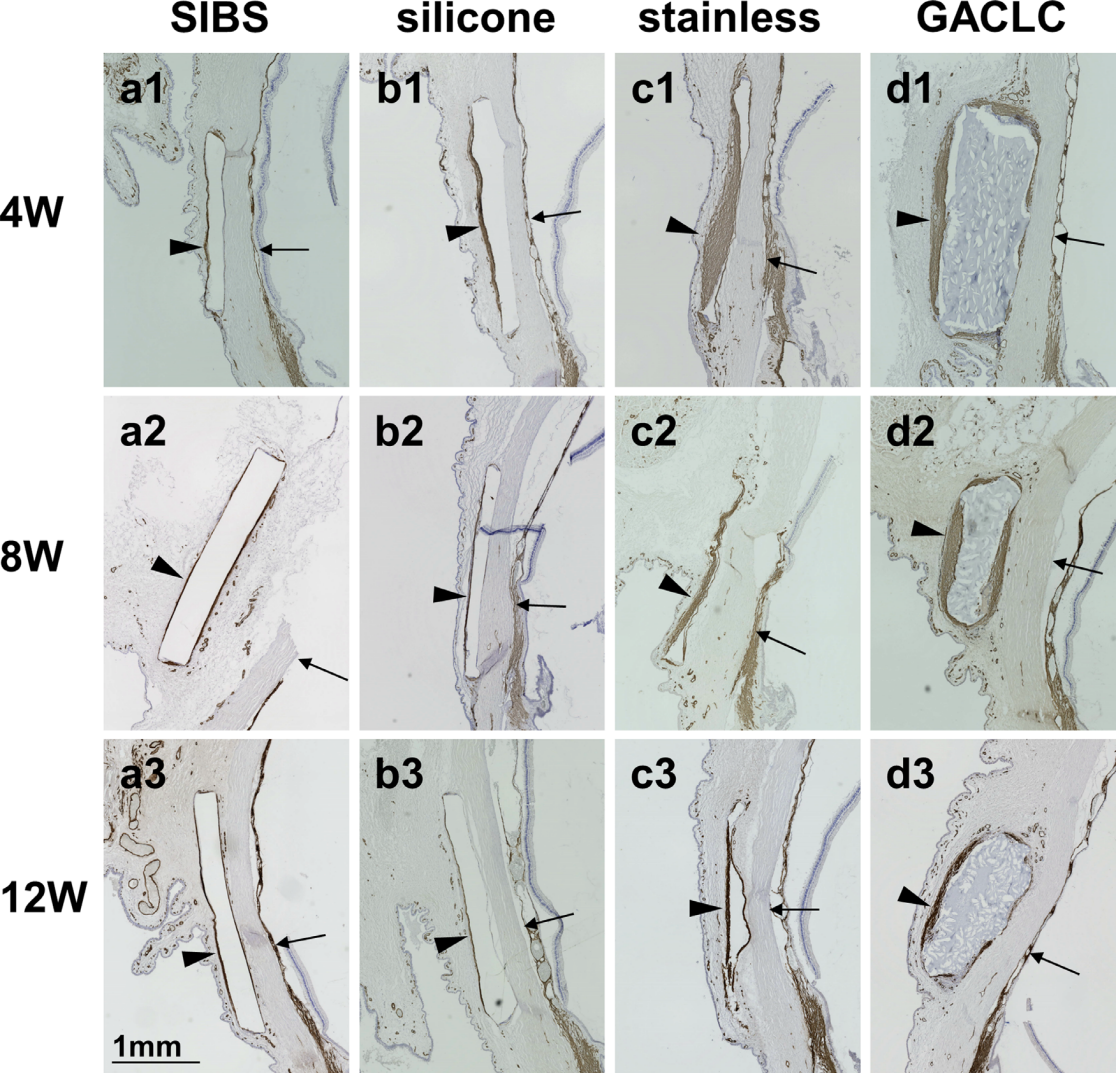
Immunohistochemical analyses of α-SMA. α-SMA antibody-immunostained images of the conjunctiva at the disk-insertion sites are shown. The columns show the eyes of the SIBS (a1–a3), silicone (b1–b3), stainless-steel (c1–c3), and GACLC (d1–d3) groups. The rows show the eyes at 4, 8 and 12 weeks after the operation. The *black arrowheads* indicate the maximum thickness of the α-SMA–positive part of the conjunctiva, and the *black arrows* indicate the sclera vertical to the same part.

**Figure 4.**
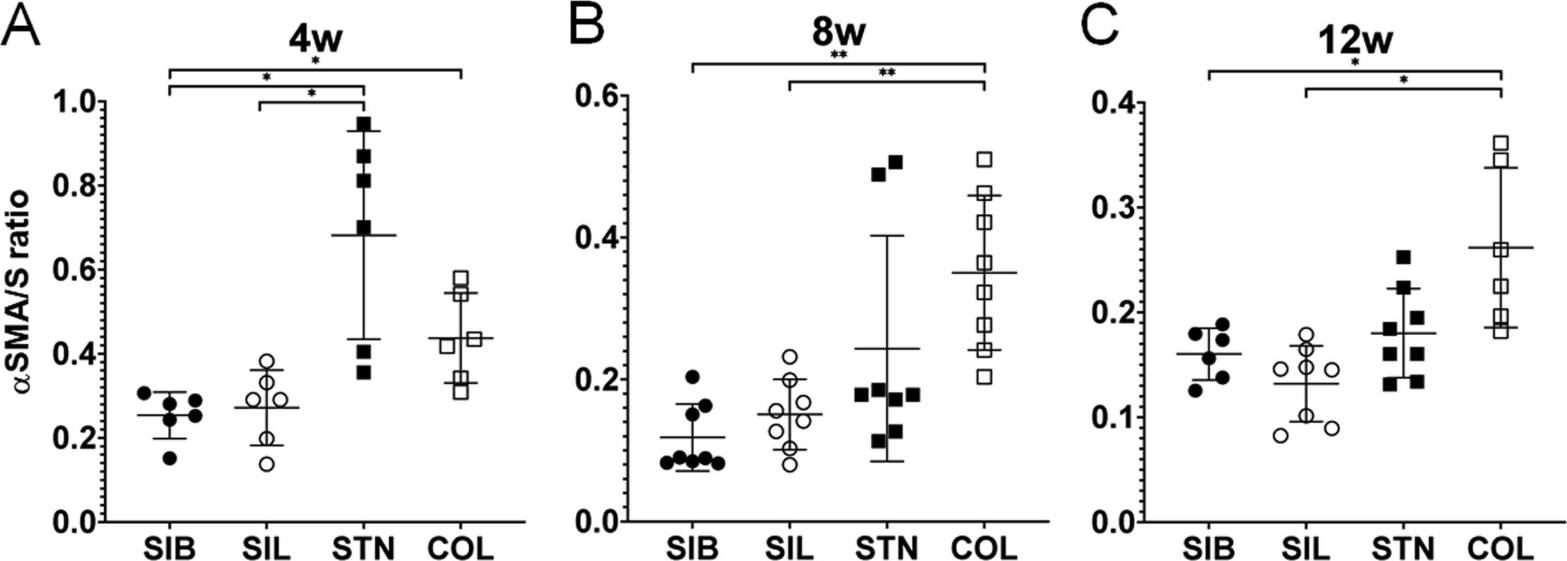
Thicknesses of α-SMA–positive areas. The ratios of the maximum thickness of the α-SMA-positive conjunctiva to the scleral thickness (α-SMA/S ratio) were calculated from immunostained images of the conjunctiva at the disk-insertion sites. Data are expressed as mean ± standard deviation. The numbers of samples in each group was six at 4 weeks and eight at 8 weeks. At 12 weeks, there were six samples in the SIB and COL groups and eight in the SIL and STN groups. *P* values were calculated using the Steel–Dwass test. *P < 0.05; **P < 0.01. SIB, SIBS; SIL, silicone; STN, stainless-steel; COL, glutaraldehyde cross-linked collagen.

**Figure 5.**
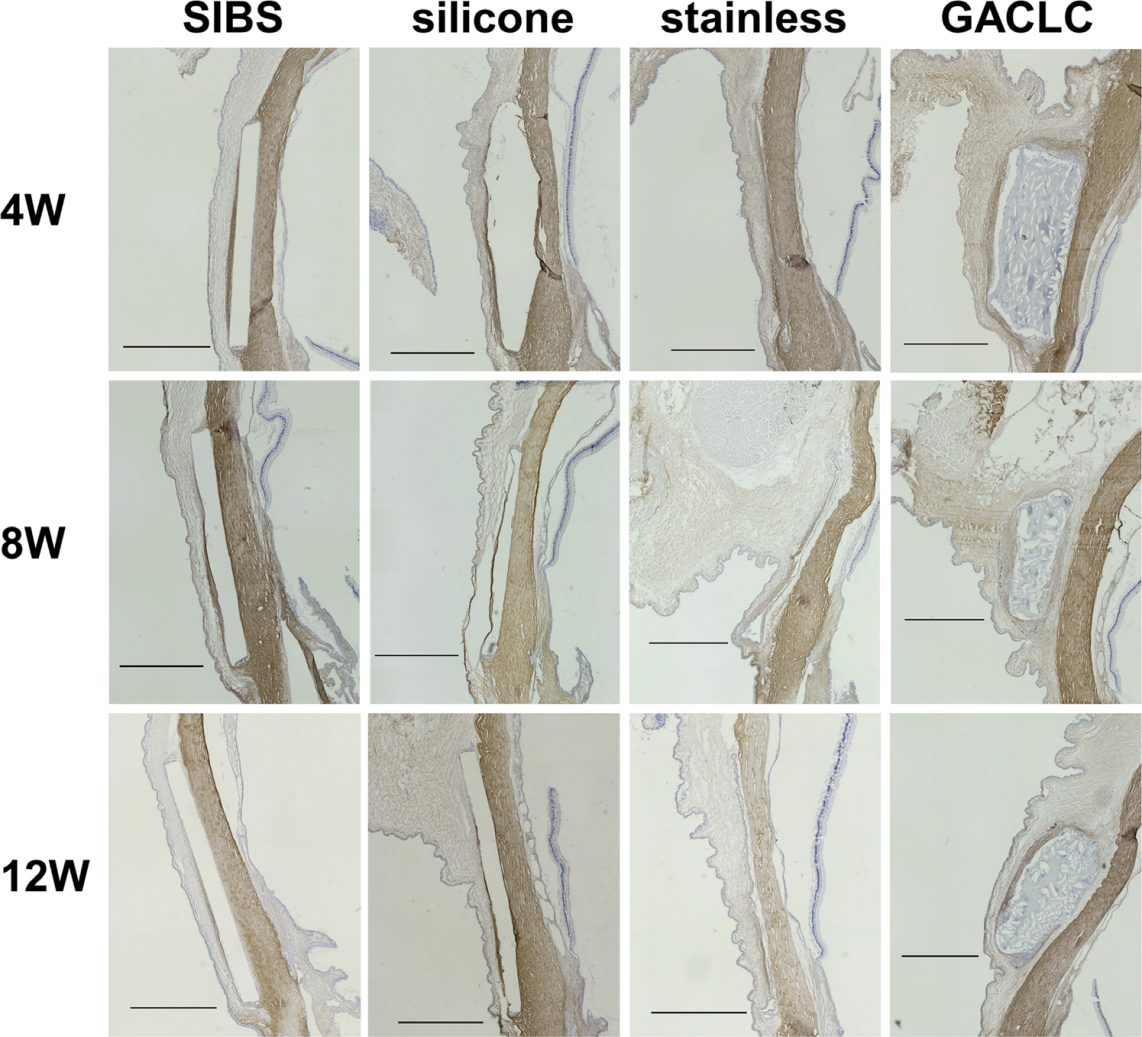
Immunohistochemical analyses of collagen type I. Immunostained images of conjunctivae at the disk-insertion sites are shown. Tissue sections were stained with collagen type I antibody. The columns show the eyes of the SIBS, silicone, stainless-steel, and GACLC groups. The rows show the eyes at 4, 8, and 12 weeks after the operation. *Scale bars*: 1 mm.

α-SMA is a marker of scarring, and we evaluated the distribution of α-SMA–positive cells at three time points (4, 8, and 12 weeks after surgery). The SIBS and silicone disks detached during slide preparation, and the stainless-steel disks were removed before slicing, so all disk-insertion sites were empty (Fig. 3). α-SMA–positive areas were observed around all disks and were thickest just outside the disks (Fig. 3). At 4 weeks after the operation, the α-SMA/S ratios were significantly lower in the SIBS group (0.254 ± 0.0505; *P* = 0.0261) and the silicone group (0.272 ± 0.0816; *P* = 0.0411) than in the stainless-steel group (0.682 ± 0.226) (Fig. 4A). The α-SMA/S ratio was significantly lower in the SIBS group than in the GACLC group (0.437 ± 0.0977; *P* = 0.0261). At 8 weeks after the operation, the α-SMA/S ratios were significantly lower in the SIBS group (0.118 ± 0.0443; *P* = 0.0074) and the silicone group (0.151 ± 0.0464; *P* = 0.0074) than in the GACLC group (0.350 ± 0.102) (Fig. 4B). At 12 weeks after the operation, the α-SMA/S ratios were again significantly lower in the SIBS group (0.160 ± 0.0227; *P* = 0.0411) and the silicone group (0.132 ± 0.337; *P* = 0.0129) than in the GACLC group (0.262 ± 0.0695) (Fig. 4C). Collagen type I–positive areas were observed around all disks (Fig. 5), particularly in the GACLC group.

### Stability of Each Material In Vivo and In Vitro

**Figure 6.**
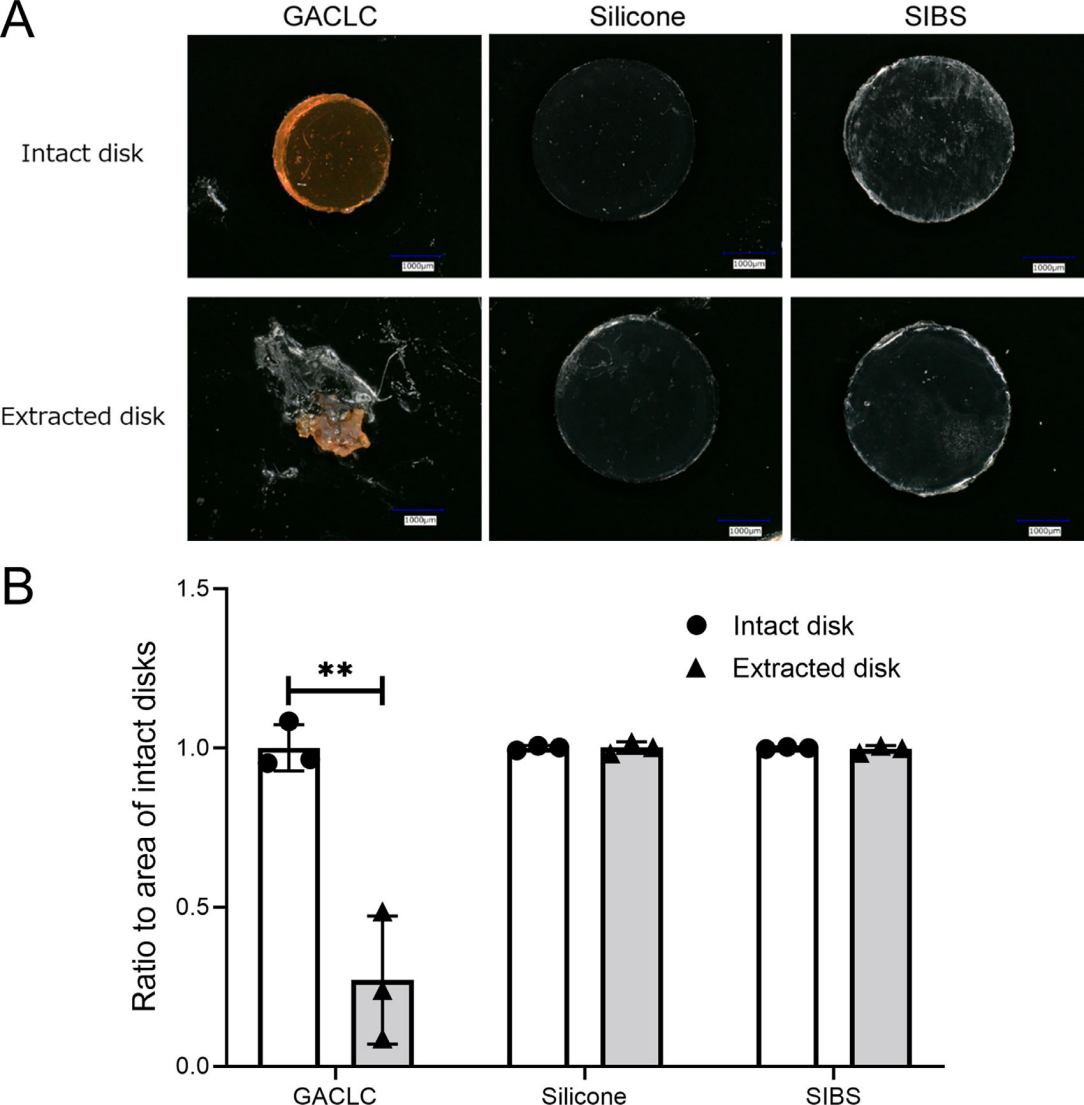
The in vivo stability of GACLC, silicone, and SIBS disks. (A) Microscopic images of GACLC, silicone, and SIBS disks are shown. The *upper* and *lower rows* are photographs taken prior to, and at 12 weeks after, insertion, respectively. *Scale bar*: 1 mm in all photographs. (B) The ratio of the surface area of intact and explanted disks. The data are expressed as mean ± standard deviation (*n* = 3). **P < 0.01, Student's *t*-test.

Microscopically, the surface areas of explanted GACLC disks were smaller after 12 weeks in vivo, but those of the silicone and SIBS disks exhibited almost no change (Fig. 6A). Although the surface areas of explanted GACLC disks were significantly smaller than those of intact disks (*P* = 0.0041), no difference was observed in the other groups (Fig. 6B).

**Figure 7.**
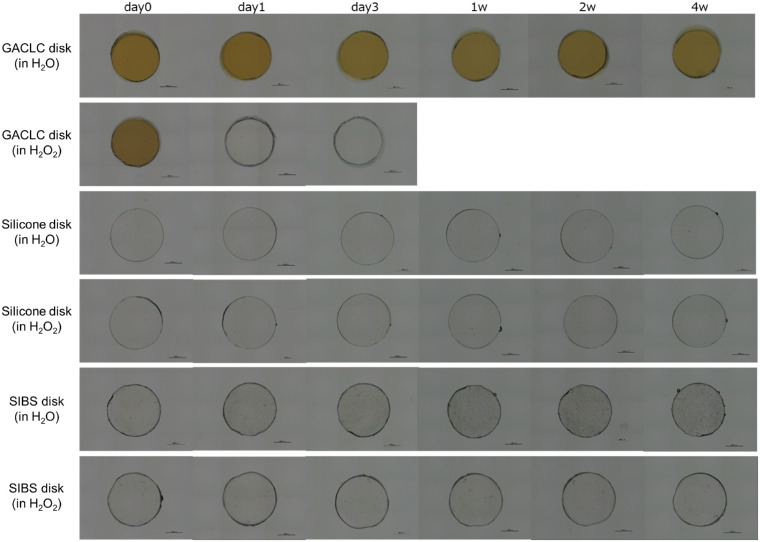
In vitro stability of GACLC, silicone, and SIBS disks. Microscopy images of the disks are shown. The rows show the time course of changes in H_2_O and H_2_O_2_ over 4 weeks. *Scale bar*: 1 mm in all photographs.

The GACLC disk color changed from yellowish to colorless after 1 day in H_2_O_2_, and the disk melted completely after 1 week, although it did not show obvious changes in H_2_O (Fig. 7). The silicone and SIBS disks did not change in either H_2_O or H_2_O_2_.

## Discussion

Although glaucoma drainage devices that reduce IOP tend to inhibit invasion and thus simplify postoperative management, IOP sometimes increases again after surgery. One possible reason is extensive scarring of the site where aqueous humor is drained and/or absorbed. The susceptibility to inflammation (and subsequent scarring, a foreign-body reaction) varies depending on the material of the drainage device.^11^^,^^17^^–^^19^ We thus explored tissue reactions to, and the stability of, four materials used to fabricate drainage devices: SIBS (the PreserFlo MicroShunt), silicone (large-plate glaucoma implants), stainless steel (the EX-PRESS Glaucoma Filtration Device), and GACLC (the XEN Gel Stent) when placed under the conjunctiva. To the best of our knowledge, this is the first study to do this.

As more hyperemia was observed in the stainless-steel group than in the SIBS and silicone groups, it was plausible that scarring caused by inflammation would be more common in the former group, and this was confirmed histologically. Fibrosis was thicker in the stainless-steel group than in the SIBS and silicone groups at 4 weeks after disk insertion. In retrospective reports of conventional trabeculectomy, laser suture lysis of the EX-PRESS mini-shunt was required earlier and more often than in trabeculectomy patients.^20^^–^^22^ Although the difference is not attributable only to a tissue reaction to stainless steel, ophthalmologists placing an EX-PRESS mini-shunt may need to observe the patient more often and prescribe additional treatment.

In our GACLC group, the disk dissolved in the rabbit conjunctiva in vivo and in H_2_O_2_ solution in vitro, and the conjunctival fibrosis was thicker than in the SIBS group at 4, 8, and 12 weeks and thicker than in the silicone group at 8 and 12 weeks. An earlier case report described XEN stent degradation at 3 years after implantation.^23^ Although this partly accords with our results, the earlier report of degradation involved the scleral and cameral regions; the subconjunctival stent region was normal. The difference may have been due to application of mitomycin C in the previous report, the presence of a bleb, and differences in the aqueous humor dynamics.

In a case series in which XEN Gel Stents and PreserFlo MicroShunts were observed for 2 years after implantation, postsurgical micropulse transscleral cyclophotocoagulation was required more often by the former group (eight of 41 vs. one of 41 eyes). Although the difference was not statistically significant, the XEN group required more needling procedures than did the MicroShunt group (eight of 41 vs. two of 41 eyes).^24^ The median time to the first needling was 2.3 months (range, 0.8–6.0) in the XEN group and 3.0 months (range, 2.1–5.1) in the PreserFlo MicroShunt group. Thus, patients who received the XEN Gel Stents tended to require more additional procedures compared with the PreserFlo MicroShunt recipients, and the difference in surgical results may have been partly attributable to a difference in tissue reactions, as observed in the present study.

Silicone has found many medical uses; it is highly biocompatible and triggers a smaller inflammatory response than the polypropylene used in some glaucoma drainage devices.^17^^,^^18^ This favorable stability was also apparent in the present study. Furthermore, we confirmed that SIBS, which is biocompatible and biostable, caused hardly any conjunctival inflammation or scarring. One study has suggested that SIBS caused minimal damage to tissues such as the conjunctiva because it is more thermoformable than silicone.^11^ Thus, SIBS may be the optimal material for fabrication of glaucoma drainage devices.

Our study had certain limitations. Although the materials were those used in commercial drainage devices, they may not have been identical. In addition, the size and structure of real drainage devices obviously differ from the disks of material used in this study. Finally, we did not assess aqueous humor dynamics, blebbing, or the use of antifibrotic agents, all of which may affect scarring. Thus, our results may not be fully generalizable to humans.

We analyzed four materials used to fabricate glaucoma drainage devices in terms of conjunctival scarring. SIBS and silicone did not induce excessive scarring or degradation, in contrast to GACLC. Further research is required to understand the properties of materials for clinical use.
